# Patterns of prescribing in primary care leading to high-dose opioid regimens: a mixed-method study

**DOI:** 10.3399/BJGPO.2022.0134

**Published:** 2022-11-30

**Authors:** John Bailey, Sadia Bashir Nafees, Simon Gill, Lucy Jones, Rob Poole

**Affiliations:** 1 Centre for Mental Health and Society, Bangor University, Bangor, UK; 2 Betsi Cadwaladr University Health Board, North Wales, UK

**Keywords:** Opioid analgesics, Drug prescriptions, Chronic pain, Primary healthcare, General practice, Mixed methods

## Abstract

**Background:**

There are concerns about continuing increases in the number of patients prescribed long-term opioids and the prescribing of 'strong' opioids for chronic pain. Little is known about patients who are prescribed these long-term, high-dose drugs.

**Aim:**

To understand patterns of opioid prescribing that lead to long-term, high-dose use.

**Design & setting:**

A mixed-method study of the opioid prescription histories of patients using high doses in a North Wales GP practice.

**Method:**

All patients on high-dose opioids during the census week were identified. Summary graphs of the prescription histories were prepared. Qualitative analysis was conducted individually by four researchers. A workshop was held to arrive at a consensus about common features and to inform further quantitative analysis.

**Results:**

A quarter of high-dose regimens were initiated outside the practice, either in a different primary care practice or in secondary care. The majority of the remaining patients showed a pattern of dose increases to high levels over a short period (median 3.5 months). None showed a pattern of gradual increases over a longer timescale. Most of the patients remained on high doses continuously once a daily dose of ≥120 mg oral morphine equivalent (OME) was reached.

**Conclusion:**

These findings suggest that high-dose opioid regimens develop quickly in response to unknown clinical factors. An expected insidious upward drift in dose was not seen. The findings have implications for the prevention of potentially dangerous long-term, high-dose opioid prescribing. A dose of 60 mg OME or more is suggested as a useful 'red flag'.

## How this fits in

There is a general assumption that high doses of opioids prescribed for chronic pain increase to those levels gradually over a prolonged timescale. This detailed analysis of individual prescription data indicated that, for most patients prescribed high doses, increases to those levels occurred within a few months. This suggests that there is a window of opportunity to prevent the development of drug regimens that are likely to be ineffective, increase risk of harm, and be maintained for years.

## Introduction

There has been a large increase in prescribing of opioids in the UK since 1990.^1–3^ Prescribed opioids are an issue of widespread concern in the UK, with focus on avoiding the severe problems encountered in the USA.^4,5^ Most opioid prescribing is for chronic non-cancer pain^6–8^ despite a lack of evidence of effectiveness for this indication^9,10^ and evidence of the risk of harms from using these drugs.^10–12^ There is epidemiological evidence that shows that opioids for chronic non-cancer pain are associated with worse functioning and quality of life,^13–16^ and clinical experience is that this is particularly prevalent in patients on high doses.

Although overall prescribing may have begun to decrease, rates are still much higher than they were 25 years ago.^2,7,17^ Of greater concern is the increased prescribing of stronger opioids^2,8,18,19^ and the increase in numbers of people prescribed them long-term,^17,20^ which might be lost sight of in any downturn in total numbers of prescriptions or items issued. Little is known about patients who are prescribed high doses of opioids long-term, who could be considered to be unlikely to benefit from their medication while being at greatest risk of harm.

Much of the existing evidence about problematic opioid prescribing comes from large, aggregated datasets. This programme of research has focused on individual level data, which allows a detailed understanding of aspects of doctor–patient interaction and prescribing that is lost in epidemiological studies. The authors have previously described a practical method to identify patients on high-dose regimens in primary care.^21^ This study examined prescribing patterns associated with high-dose regimens.

Prevailing clinical opinion led to an initial hypothesis that high-dose regimens resulted from a gradual process consisting of a series of dose increases interspersed with periods of stable prescribing, with an assumption that increases were driven by drug tolerance.

## Method

### Study setting

This study is a based on data from a longitudinal study of opioid prescribing in a large primary care centre in North Wales. The practice is in a rural market town with social deprivation indices close to the mean for Wales.^22^ Local Health Board prescribing data show that, during the period of interest, the level of opioid prescribing in the practice was below the mean for North Wales. The Local Health Board had opioid prescribing levels below the mean for Wales.

### Data collection

Using the method previously described,^21^ patients prescribed high doses of opioids for pain were identified and detailed opioid prescription histories obtained from practice records. The census point was the week beginning 8 January 2018 and the criterion for high-dose opioid use was a daily dose of ≥120 mg of OME medication (see Table 1). The following data were collected for every opioid item prescribed in the practice to patients meeting this criterion since records were computerised in 1997: the prescription date; the drug prescribed; its strength and formulation; the amount prescribed; the dose direction; and the prescriber.

**Table 1.** Doses equivalent to 120mg/day of oral morphine. Calculated using the 'traditional' conversion tables produced by Palliative Care Guidelines Plus.^23^

Drug	Dose
Oxycodone	60 mg/day
Fentanyl^a^	33.3mcg/hr
Buprenorphine^a^	50mcg/hr

^a^All fentanyl and all relevant buprenorphine was prescribed as transdermal patches.

The analysis of prescribing patterns was challenging. Each patient who met the study criteria had typically been prescribed several hundred items. This study was interested in how doses increased: given the numbers involved and the heterogeneity of timescales, the authors decided to use a novel method to examine the prescribing histories. This was a mixed-method analysis in three stages that relied on individual judgement to identify patterns and common features in graphs generated from the quantitative data (see Supplementary file).

### Data preparation for initial quantitative data analysis

Summary graphs were prepared for each patient that plotted daily OME opioid doses over time. All changes in prescribed OME dose were charted, in addition to the dose prescribed at the beginning and mid-point of each year irrespective of whether a change had occurred. Each chart also gave information about the opioid drug(s) prescribed and the age and sex of the patient. Where there was a direction for PRN (pro re nata) use, the daily dose was estimated (by dividing the amount prescribed by the number of days between prescriptions) and included in the total OME dose. The daily dose was also estimated where there was a clear discrepancy between the amount prescribed and the dose direction (for example where the dose directed was for a 4-week period but prescriptions were being issued at shorter intervals); otherwise the directed dose was plotted.

### Qualitative data analysis

There were four assessors of the qualitative data:

- A clinical nurse specialist in pain management (LJ).
- An academic psychiatrist with an interest in pain (RP).
- A primary care pharmacist specialising in prescribing for pain (SG).
- A researcher investigating the prescribing of opioids for pain (JB).

Each was provided with a complete set of the graphs and given the instructions listed in Box 1.

Box 1 **Instructions to the assessors**

  You should have 32 graphs and a key.

These represent the opioid prescription histories of all the patients with doses of 120mg OME or more in our 2018 audit in ***** practice.

Our general aim is to make sense of these graphs. You are asked to carry out three tasks.

1. Examine the graphs, individually and collectively, and note any observations you have.
2. Organise the graphs into groups, on the basis of some feature shown in the graph, noting the members of the group and the criteria for group membership. For each set of criteria there should be two groups – those which meet the criteria and those who do not. Do this as many times and in as many different ways as you think useful.
3. Note any queries raised by the data and any further comments, indicating which patient or group(s) they refer to.

  Please record your notes, comments, group criteria and members, etc. in Word file(s).

              ……………………

A meeting will be arranged, chaired by SN, for the participants to identify common themes, discuss and resolve differences and arrive at a consensus view of the meanings which can be extracted from the data.

Responses were collated and a digest of the comments and questions prepared. This informed a workshop of the assessors (chaired by an experienced qualitative researcher; SN) where common themes were identified, differences discussed and resolved, and a consensus view of the meanings that could be extracted from the data was reached.

On the basis of the conclusions of the consensus workshop, there was a second quantitative data analysis.

## Results

### Initial data analysis

There were 32 patients who met the 120mg OME criterion for high-dose use of opioids at the census point and for whom opioid prescription histories could be obtained from practice records. Eight of these patients had high-dose use initiated elsewhere: records showed three were being prescribed these doses when they joined the practice and indicated that five had doses initiated in secondary care.

### Qualitative data analysis

At the consensus workshop, it was agreed that a major feature of the qualitative data was that increases in doses suggested a particular pattern of high dose development and that further analysis of the prescription data was required. Three main areas were agreed to require further investigation.

1. Increased prescribing that led to doses above the high-dose criterion. The qualitative data suggested that many of these increases took place over a short timescale.
2. When high doses were reached, these appeared to be sustained in many of the patients.
3. The length of time opioids had been prescribed before the increase to high doses occurred.

The disproportionate role of fentanyl in high-dose prescribing was noted, as was the number of older patients being prescribed high doses of opioids.

### Further quantitative analysis

**Figure 1.**
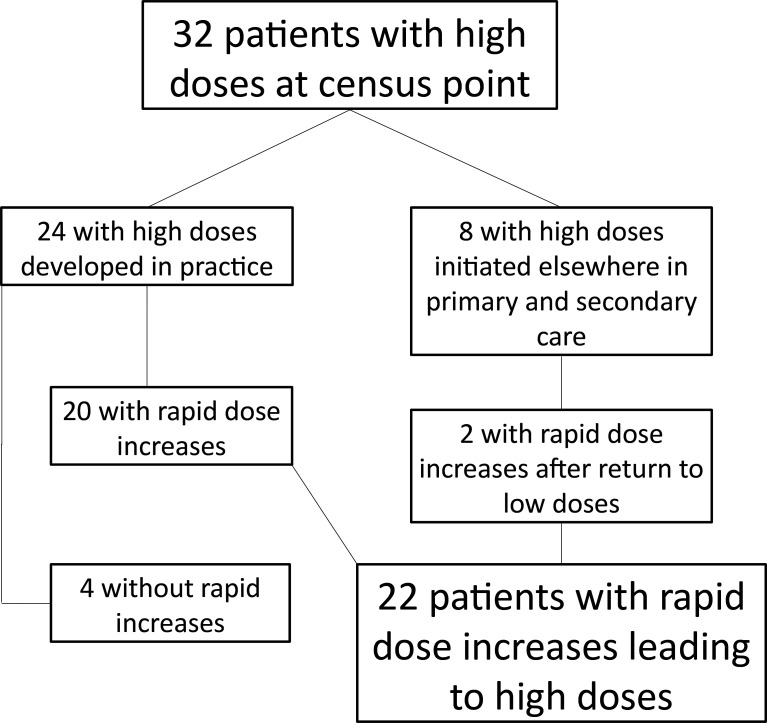
Identification of patients with rapid dose increases

A dose increase of ≥90mg OME that took place within 1 year and which resulted in a daily dose of ≥120mg OME was set as the criterion for the phenomenon of interest. Only four patients did not have prescribing episodes that met this criterion. Two of the patients with high doses initiated in secondary care had prolonged high doses followed by a period of much reduced doses and a second episode that met the rapid increase criterion. (See Figure 1.)

Of the 22 patients with rapid dose increases meeting the criterion, 15 had increases that met the criterion in less than 6 months. For three of the 15, the increase was immediate (between one prescription and the next). The increases of the remaining seven met the criterion between 6 months and 1 year. The median length of time of the increases was 3.5 months. The median dose increase was 162mg OME. Episodes of rapid dose increase occurred over 14 years and involved 17 different prescribers.

For 11 patients the main opioid involved in the rapid increase was fentanyl, including all three patients with immediate increases; for seven it was oxycodone; for three it was morphine; and for one it was buprenorphine. Eighteen of the 22 patients were prescribed a daily dose of ≥60mg OME of one opioid, either at the beginning or in the early stages of the increase. Of the 32 patients on high doses of opioids at the census point, 16 were on fentanyl, 10 were on oxycodone, five on morphine, and one on buprenorphine.

For nine patients, rapid increases began within a year or less of being prescribed a first 'strong' opioid (fentanyl, oxycodone, or morphine) and a further six within 2 years of a first 'strong' opioid.

Two of the 22 patients with rapid increases met the criterion close to the audit point and were, therefore, not included in assessments of sustained high doses. Fifteen patients were prescribed doses that, after dose increase, did not at any point drop below the high-dose criterion. Two of the remaining five patients had a single brief period of prescribing below the high-dose criterion.

At the census point, four patients had been prescribed continuous high doses for 10 years or more, and a further eight for 5 years or more.

Fourteen of the patients on high doses at the census point were aged ≥65, and of these seven were over 80.

## Discussion

### Summary

None of the patients in this study showed the initially hypothesised pattern of dose increases. Of the 26 patients for whom data on the prescription histories leading to high-dose regimens were available, 22 showed large increases in doses over a short timescale. The remainder had slower dose increases, but none showed the expected staged increase pattern. In addition, it is likely that the patients whose high-dose regimens originated in secondary care also experienced similar rapid dose increases. This suggests that increase are unlikely to be a response to slowly emerging opioid tolerance.

This is an analysis of the opioid prescription histories of a cross-sectional sample of primary care patients prescribed high-dose opioids. It indicates a critical point in those histories when prescribed doses increased rapidly to levels that are likely to be both ineffective in providing additional pain relief and damaging to daily functioning and quality of life. It also indicates that once high-dose regimens are initiated, they are likely to be in place for a long time.

The qualitative analysis also raised concerns about the number of older patients with high-dose regimens and the disproportionate role of fentanyl in high-dose regimens.

### Strengths and limitations

The strength of this study is that the small scale allowed detailed case-by-case analysis of data, which would be impossible in a large-scale dataset. The use of the innovative mixed methods described here identified patterns of individual prescription histories that would not have emerged from a simple numerical analysis, and resulted in findings that are directly applicable to clinical practice rather than identifying population trends.

A weakness of small-scale studies like this one is that their data may be drawn from atypical sources and therefore subject to biases that may be difficult to detect; however, there is no obvious reason to think the GP practice in this study is atypical. Another possibility is that the results of the study are biased by unusual behaviour at a particular time or by a small number of individual practitioners; the length of time over which the phenomenon observed occurred and the number of prescribers involved suggest this is not the case here.

Investigator bias is a potential problem with the qualitative data analysis methods in this study. The consistency of the findings and that none of the cases examined exhibited the expected prescribing pattern suggest that this is not a factor here.

### Comparisons with existing literature

As noted in the Introduction, there is research that shows increasing population levels of long-term opioid prescribing and of the prescribing of higher-dose drugs. The most recent systematic review of opioid prescribing for chronic pain suggests that most harms are dose-dependent.^10^ However, the authors are not aware of any other research studies that have systematically examined the development of high-dose prescribing of opioids for chronic pain at the individual patient level.

### Implications for research and practice

These findings suggest that an identifiable opportunity exists to take action to prevent ineffective and harmful high-dose regimens from developing by reviewing cases, by exploring alternative treatment strategies, and through the vigilant management of prescribing. However, the short time over which rapid increases take place means that routine annual medication reviews may fail to detect the development of high-dose regimens. This study's data suggest that the prescribing of an opioid dose of ≥60mg OME of a single drug would be a useful 'red flag'.

There is a need for the replication of these findings, in other places and using other methods, given their divergence from generally accepted expectations.

## Data

The dataset relied on in this article is available from the corresponding author on reasonable request.
